# Efficacy and safety of Jianpishengsui for chemotherapy-related fatigue in patients with non-small cell lung cancer: study protocol for a randomized placebo-controlled clinical trial

**DOI:** 10.1186/s13063-019-3982-3

**Published:** 2020-01-16

**Authors:** Zhiwei Xiao, Leihao Hu, Jietao Lin, Liming Lu, Xuewu Huang, Xiaoshu Zhu, Chiahshean Teo, Lizhu Lin

**Affiliations:** 1 Oncology Center, The First Affiliated Hospital of Guangzhou University of Chinese Medicine, Guangzhou, 510405 Guangdong China; 2 Guangzhou University of Chinese Medicine, Guangzhou, 510405 Guangdong China; 3 Clinical Research Center, South China Research Center for Acupuncture and Moxibustion, Medical College of Acu-Moxi and Rehabilitation, Guangzhou University of Chinese Medicine, No.232 Waihuan Dong Road, Guangzhou, 510006 Guangdong China; 4 School of Science and Health,Chinese Medicine Centre, Western Sydney University, Penrith, NSW Australia; 5 Traditional & Complementary Unit, National Cancer Institute, 4, Jalan P7, Presint 7, 62250 Putrajaya, Malaysia

**Keywords:** Chemotherapy-related fatigue, Cancer-related fatigue, Non-small cell lung cancer, Jianpishengsui, Herbal formula cream

## Abstract

**Background:**

Chemotherapy-related fatigue (CRF) is a common symptom in non-small cell lung cancer (NSCLC) patients. A Chinese herbal formula cream for oral application, called Jianpishengsui (JPSS), is extensively used in the First Affiliated Hospital of Guangzhou University of Chinese Medicine as an internal preparation for CRF and is associated with a promising response. Due to the lack of high-quality clinical evidence, a randomized placebo-controlled trial is required to assess the efficacy and safety of JPSS.

**Methods/design:**

The efficacy and safety of JPSS herbal formula cream will be evaluated through a prospective, randomized, placebo-controlled trial conducted in the First Affiliated Hospital of Guangzhou University of Chinese Medicine. NSCLC patients with CRF will be randomized into two groups at a ratio of 1:1. Each group will receive either 15 g of the oral JPSS herbal formula cream or placebo twice a day from day 6 to day 20 during two courses of paclitaxel + platinum/docetaxel + platinum/pemetrexed + platinum (TP/DP/AP) chemotherapy. The primary endpoint is the difference in the degree of fatigue between baseline (the day before the start of the intervention) and day 42, which will be assessed by the Revised Piper Fatigue Scale score. The secondary endpoints are quality of life (measured by the 43-item European Organization for Research and Treatment of Cancer Quality of Life Questionnaire—Lung Cancer C43), Eastern Cooperative Oncology Group Performance Status, and Traditional Chinese Medicine syndrome score. The toxicity of the treatments will also be evaluated at the same time. All outcomes will be measured at baseline, day 6, day 21, and day 42 of the treatment.

**Discussion:**

This randomized trial will investigate the efficacy and safety of JPSS applied for CRF in patients with NSCLC.

**Trial registration:**

Chinese Clinical Trial Registry, ChiCTR1900023451. Registered on 28 May 2019.

## Introduction

Lung cancer is a major factor contributing to cancer-related death worldwide. It is estimated that non-small cell lung cancer (NSCLC) accounts for approximately 85% of lung cancer incidence [1]. Most NSCLC patients are diagnosed at an advanced stage, which leads to a very large reliance upon systemic chemotherapy. It is estimated that 80–96% of patients suffer from chemotherapy-related fatigue (CRF) [2, 3]. CRF has a profound negative impact on the patients’ quality of life. Patients usually have symptoms such as loss of appetite, dry mouth, anxiety, tension, nausea, and insomnia. These symptoms may last for a long period and cannot be alleviated by rest or sleep, leading to treatment interruption or delay amongst patients. CRF belongs to the category of cancer-related fatigue, which is mainly experienced by patients undergoing chemotherapy [4].

In recent years, scholars have noted that the potential pathogenesis of CRF may be related to anaemia [5], abnormal regulation of cytokines [6, 7], abnormal regulation of hypothalamic–pituitary–adrenal axis function, [8] dysrhythmia, [9] and skeletal muscle atrophy [10]. Chemotherapy-induced anaemia is considered one of the leading causes of CRF [11]. However, not all patients who experience exhaustion have anaemia, and most patients continue to feel weariness after recovery. At present, no standard therapies are available for treating CRF. Physical exercise, such as Tai Chi, yoga, family sports, nursing guidance, physical therapy, and psychosocial intervention, has been widely recognized as an effective method to overcome CRF [12, 13]. However, patients with CRF, especially those with a severe index, are usually too weak to perform traditional exercise. While the underlying mechanism of CRF remains unclear, there is currently no recognized safe and low-toxic specific drug for its treatment [14]. Previous studies have focused mostly on the use of haematopoietic growth factors, psychostimulants, dexamethasone, or antidepressants to treat CRF, but the results have been mixed [15–18]. Alternatively, traditional Chinese medicines, such as acupuncture [19] and herbal extract formula creams, have become a treatment option for Chinese patients. The herbal extract formula cream is widely used for the prevention and treatment of chronic diseases in China [20–22].

According to the theory of Chinese medicine, both Qi and blood are basic and vital components of the human body and can maintain activities of living. Qi [23] refers to the energy flow of the body (or a vitality of the body), which maintains blood circulation, warms the body, and fights against disease. In Traditional Chinese Medicine (TCM), blood deficiency [24] is considered a pathological state of blood dysfunction and organ dystrophy, which is often caused by spleen and stomach deficiency, haematopoiesis, and blood stasis. Qi and blood supplement each other and support vigour. Qi and blood deficiency are the main pathogenic factors involved in CRF [25]. Qi and blood deficiency syndrome are commonly accompanied by fatigue, shortness of breath, decreased activity, poor sleep, and loss of appetite.

Based on the clinical experience of Professor Lizhu Lin, a well-known TCM oncologist, we developed an internal preparation called Jianpishengsui (JPSS) herbal formula cream in the First Affiliated Hospital of Guangzhou University of Chinese Medicine. JPSS has been the most commonly used formula to treat CRF for the past 4 years (hospital preparation approval number Z20151106). A previous study showed that JPSS has a good effect on patients with CRF, and no adverse reactions have been reported. Yu Ling [26] conducted a prospective, randomized, controlled study to assess the effectiveness of JPSS on cancer-associated anaemia. One hundred and eleven patients were randomly divided into the experimental group (51 cases) or the control group (60 cases). The experimental group was treated with JPSS combined with erythropoietin for a total of 42 days, while the control group was treated with erythropoietin alone. The results showed that the RBC and Hb levels were significantly improved in the experimental group, while the Piper scores were significantly reduced (*p* < 0.05), which indicated that JPSS might have a remarkable influence on CRF [26]. JPSS is a mixed Chinese herbal formula cream consisting of 17 different herbs, of which *Codonopsis pilosula* and *Carapax Trionycis* are the main components. Each of these herbs can synergistically nourish Qi and blood, and relieve fatigue to a certain extent. Syndrome differentiation is the core concept of TCM. For this reason, we chose patients with both Qi and blood deficiency as the target population.

In comparison with the traditional decoction, the herbal formula cream is easy to use because it can be stored in the refrigerator and taken with water. Previous studies have found that JPSS has a good effect on fatigue caused by cancer-related anaemia with few side effects. We are also applying for a patent for the JPSS herbal formula cream. Despite its extensive clinical use, the safety and efficacy of JPSS for treating CRF have not been investigated in a prospective, randomized clinical study. Therefore, there is a need to conduct a JPSS intervention study for NSCLC patients with moderate-to-severe fatigue.

## Methods/design

### Design and setting

**Fig. 1:**
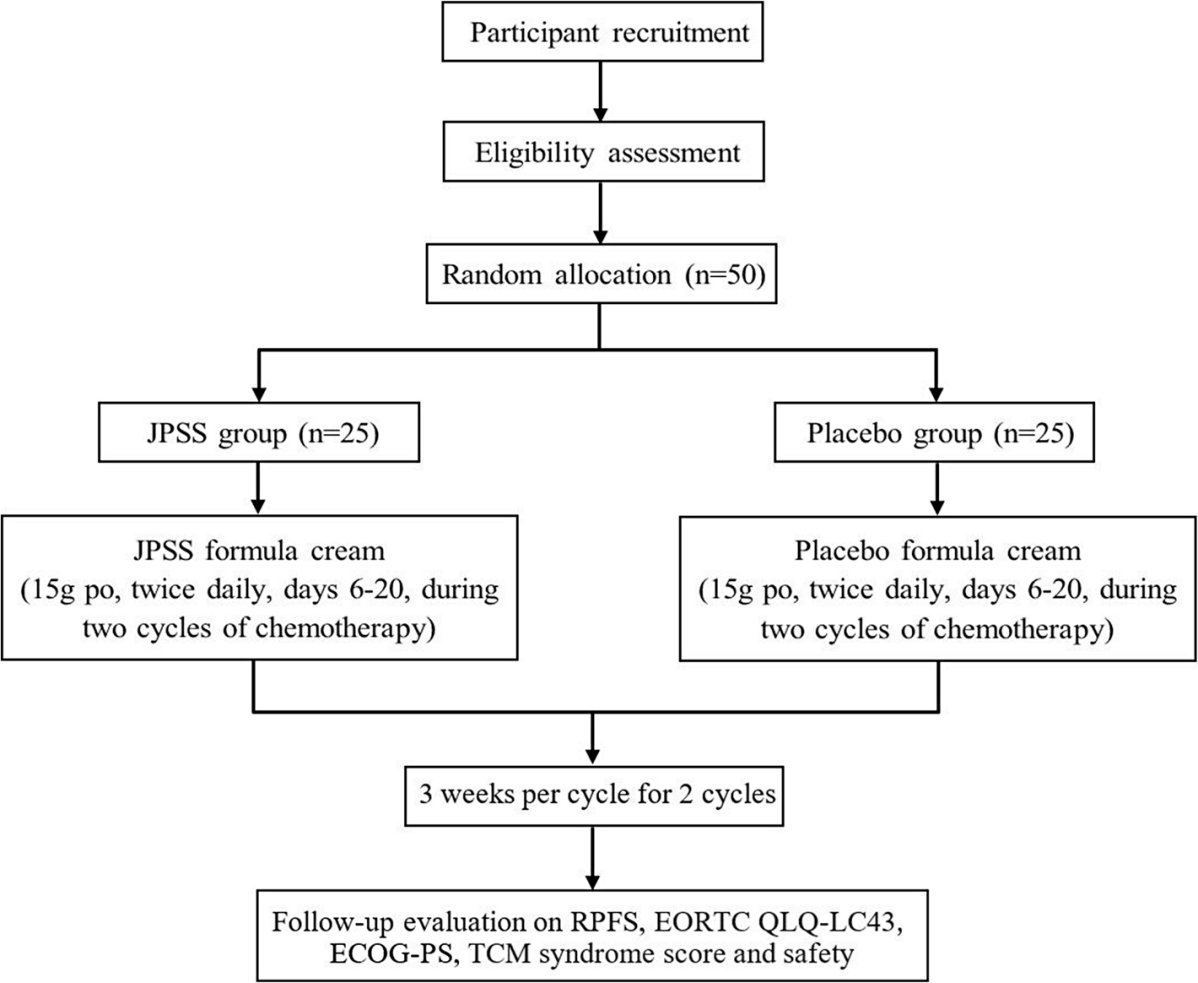
Flow chart of the trial procedure. ECOG-PS Eastern Cooperative Oncology Group performance status, EORTC QLQ-LC43 43-item European Organization for Research and Treatment of Cancer Quality of Life Questionnaire C43, JPSS Jianpishengsui, RPFS Revised Piper Fatigue Scale, TCM Traditional Chinese Medicine

A prospective, randomized, placebo-controlled trial, as shown in Fig. 1, will be performed on NSCLC patients in a single centre during the advanced stage of CRF. Fifty patients with deficiency of Qi and blood syndrome and CRF will be selected and then randomly divided into two equal-sized groups: the JPSS group and the P group. The JPSS group will receive treatment with the JPSS herbal formula cream, while the P group will receive the placebo formula cream. Both groups will receive the JPSS herbal formula cream or the placebo from day 6 to day 20 during the two courses of paclitaxel + platinum/docetaxel + platinum/pemetrexed + platinum (TP/DP/AP) chemotherapy.

This trial has been approved by the Ethics Committee of the First Affiliated Hospital of Chinese Medicine (No. ZYYECK[2018]045).

### Participants

The project will be carried out in the First Affiliated Hospital of Guangzhou University of Chinese Medicine by recruiting patients from either outpatient or inpatient settings.

Participants will be recruited through advertisements and referrals from 1 July 2019 to 31 November 2019. Advertisements will include social media, posters, and flyers in hospital and public areas, such as the cancer centre and outpatient waiting halls.

Patients with a diagnosis of NSCLC (TNM stages IIIb–IV) based on pathology or cytology, a diagnosis of moderate-to-severe fatigue, and a diagnosis of Qi and blood deficiency syndrome can be enrolled in this study. The diagnostic criteria and TNM classification for NSCLC are based on the Guidelines for Diagnosis and Treatment of Common Cancers in China (Chapter of Malignant Neoplasm). The diagnostic criteria for CRF are based on the 10th revision of the International Classification of Diseases. Patient-reported fatigue severity is measured using the Revised Piper Fatigue Scale (RPFS). The diagnosis of Qi and blood deficiency syndrome criteria is based on the Guidance Principle of Clinical Research on New Drug of Traditional Chinese Medicine (2002 edition) [27].

Qi deficiency syndrome can be diagnosed on the basis of two main symptoms and one secondary symptom as follows: main symptoms include shortness of breath, lack of strength, fatigued spirit, and vacuous pulse; secondary symptoms include spontaneous sweating, short speech, and pale tongue. Blood deficiency syndrome can be diagnosed on the basis of two main symptoms and one secondary symptom as follows: main symptoms include pale white or sallow complexion, dizziness, clouded flowery vision, and palpitations; secondary symptoms include insomnia, numbness of extremities, and abnormal menstruation (e.g., deferred, scanty, or light colour of menstruation). Any patients who meet both of these criteria will be diagnosed with Qi and blood deficiency syndromes.

### Recruitment procedure

The potential participants will be contacted in outpatient or inpatient settings before enrolment. Patients who have received at least one course of treatment and no more than four courses of chemotherapy will be recruited as potential subjects. A TCM oncologist trained in TCM diagnosis and modern oncology will screen the medical eligibility of the patient. Written informed consent from all participants is required before enrolment, and patients will have sufficient time to decide whether to participate before signing the consent form. After an assessment of CRF severity and other relevant entry criteria within a week before the start of the intervention, enrolment will be confirmed, and baseline characteristics including sex, age, body mass index, pathological type, baseline RPFS, ECOG score, and disease history will be recorded. Participants will undergo blood tests, including full blood count, urine analysis, stool analysis, liver function test, and renal function test, at baseline, day 6, day 21, and day 42.

### Inclusion criteria

1. NSCLC diagnosis (TNM stages IIIb–IV) based on pathology or cytology
2. Moderate-to-severe fatigue (with RPFS score ≥ 4) after cancer diagnosis due to cancer therapy or cancer itself
3. Wild type or unknown driver gene
4. TCM syndrome differentiation in line with the diagnostic criteria of Qi and blood deficiency syndrome
5. Experience with at least one and a maximum of four cycles of chemotherapy and plan to receive chemotherapy (TP/DP/AP scheme) for at least two courses
6. Age range: 18–75 years
7. ECOG score ≤ 2
8. Voluntary participation with written informed consent
9. No use of antidepressants or other psychotropic drugs within 1 month of enrolment in the study
10. Willingness to participate in surveys and ability to complete the questionnaires independently with clear consciousness and without cognitive or psychotic disorders

### Exclusion criteria

1. Treatment with EGFR/ALK-targeted medicine or PD-1/PD-L1 immune checkpoint blockade therapy
2. Allergy to JPSS granules (including patients who have allergies to any component of the prescription); allergy to black beans or bean products
3. Patients with uncontrollable infections that require the use of anti-inflammatory drugs
4. Anaemia, defined as haemoglobin level < 90 g/L or PLT < 50 × 10^9^/L within 2 weeks from the date of enrolment
5. Thyroid disorder with abnormal thyroid-stimulating hormone and free T4 levels
6. Any significant comorbidity, such as severe insomnia or depression and reduced oral intake
7. Pregnancy or lactation
8. Severe liver or kidney dysfunction (serum creatinine ≥ 1.5 times ULN; ALT or AST ≥ 1.5 times ULN; bilirubin ≥ 1.5 times ULN) or a history of hepatitis A, B, or C
9. Current use of the following drugs: ginseng, methylphenidate, modafinil, phenobarbital, phenytoin, clonidine, or tricyclic antidepressants

### Interventions

The treatment group will receive 15 g of the JPSS herbal formula cream twice daily from day 6 to day 20 for each course of chemotherapy, for 30 days in total. The control group will receive 15 g of the placebo formula cream twice daily for 30 days in total; the main ingredients of the placebo are broken-down black soya bean, Guiling jelly, caramel colour, and Yuan Zhen sugar. The fabrication method, appearance, smell, and taste are similar to those of JPSS. The JPSS herbal formula cream and the matching placebo used in this trial are manufactured by the First Affiliated Hospital of Guangzhou University of Chinese Medicine in a way that meets the requirement of the Good Manufacturing Practice (GMP).

Patients will take the cream orally on their own and receive instructions. Customized spoons and measuring cups will be provided. The using method of JPSS: directions—put 15 g of JPSS into a cup, stir it with warm water, and take it twice a day after breakfast and dinner; storage—away from light below 20 °C is recommended; and any discomfort while taking the medication should be reported to the researcher using the published telephone number.

All patients will receive JPSS or placebo during two courses of platinum-based chemotherapy. The specific scheme and dosage were established according to the US National Comprehensive Cancer Network (NCCN) clinical practice guidelines in oncology. The recommended platinum-based chemotherapies are the TP/DP/AP scheme. A research nurse will observe every patient once a week. Follow-up information on adverse effects, compliance, and combined use of other medicines will be collected through interviews, telephone calls, and questionnaires.

The Jianpishengsui (JPSS) herbal formula cream is one of the most commonly used hospital internal preparations. The recipe is an empirical prescription from Professor Lizhu Lin. The herbal formula cream is made of multiple Chinese medicines, and the main ingredients include the following:

- *Carapax Trionycis*
- *Cervus nippon Temminck*
- *Codonopsis pilosula*
- *Lycium chinense Miller*
- *Polygonatum sibiricum*
- *Fructus Ligustri Lucidi*
- *Herba Ecliptae Eclipta prostrala L*
- *Pericarpium Citri Reticulatae*
- *Endothelium Corneum Gigeriae Galli*

Preparation of the JPSS is as follows: soaking (add the ingredients to a specific decocting bag, add cold water in an amount 10 times that of the herb, and continuously soak the ingredients in the decocting machine for 12 h); decoction (first, perform the first decoction for 2 h and then filter the solution; second, add six times the amount of water to the herbs for 1 h for the second decoction, and then filter the solution; and third, mix the two solutions); concentration (pour the mixed solution into a high-pressure vacuum concentration decocting machine to concentrate it under constant temperature (~60–70 °C) until it turns into a light cream); and collecting the cream (transfer the light cream to a thermostatic capacitor to continue to concentrate it until there is no vapour; then, collect and package the herbal formula cream). A box of JPSS weighs 500 g and is recommended to be stored away from light at a temperature below 20 °C.

### Randomization

We will use the minimum randomization method to perform randomization. Eligible patients will be randomized into the JPSS group and the placebo (P) group in a 1:1 ratio for a target total of 50 patients. Patients will be randomized by the Statistical Analysis System (SAS 9.4 software). The process will be performed by an independent statistician in the Clinical Research Center, South China Research Center for Acupuncture and Moxibustion, Guangzhou University of Chinese Medicine.

### Blinding

Only the independent statistician will know the group situation and have access to the randomization list and blinding codes. The independent statistician will label the cream according to the blinding codes and then tear off the marking label that distinguishes the placebo from the JPSS cream. He will not be involved in the outcome data analysis. The research pharmacy is responsible for the distribution of the cream. This study will be a double-blind trial in which both the participants and researchers, including nurses, physicians, and analysing statisticians, are blinded to the allocated treatment.

The randomization schedule will be hidden until all interventions are assigned and registration, follow-up, data collection, data cleansing, and analysis are completed. Blinding will be ensured using a placebo of the same colour, size, shape, and taste. The quality of the test product, such as the content, solubility, and bacterial contamination, will be strictly controlled by GMP standards and tested and verified by the researchers.

### Outcome measurements

The primary outcome is the difference in the Revised Piper Fatigue Scale (RPFS) score compared in the two groups between baseline (the day before the start of the intervention) and day 42. Secondary outcome measures include quality of life (QoL), Eastern Cooperative Oncology Group Performance Status (ECOG-PS), and TCM syndrome scores. Patients’ demographic data, including age, sex, body mass index, histopathological type, and past medical history, will be recorded at the time of entry into the study. The patients’ safety will be monitored throughout this study.

#### Revised Piper Fatigue Scale

The Revised Piper Fatigue Scale (RPFS) is a self-rating scale and a multidimensional assessment tool that has been widely used to measure CRF [28]. Four dimensions are encompassed in the RPFS, including behavioural/severity (six items), affective meaning (five items), sensory (five items), and cognitive/mood (six items). There are a total of 22 items in the RPFS. Every item is scored on a scale of 0–10, with 0 representing no fatigue, 1–3 representing light levels of fatigue, 4–6 representing mild levels of fatigue, and 7–10 representing high levels of fatigue. The more severe the fatigue, the higher the score will be. Researchers will assess the RPFS score at baseline, day 6, day 21, and day 42.

#### EORTC QLQ-LC43

The European Organization for Research and Treatment of Cancer Quality of Life Questionnaire—Lung Cancer 43 (EORTC QLQ-LC43) is an instrument for assessing QoL in lung cancer patients. The EORTC QLQ-LC43 contains the EORTC QLQ-C30 and the EORTC QLQ-LC13 [29]. The former involves five functional domains (physical, cognitive, emotional, role, and social), three symptomatic domains, one overall quality-of-life domain, and six single domains. The latter involves 13 specific items for lung cancer. Their reliability, validity, and responsiveness have been verified in China [30]. The detailed scoring method will refer to the literature. The evaluation will be performed at baseline, day 6, day 21, and day 42.

#### ECOG-PS

The Eastern Cooperative Oncology Group Performance Status (ECOG-PS) is widely used in clinical practice to assess a patient’s general condition. The evaluation will be performed at baseline, day 6, day 21, and day 42.

#### TCM syndrome scores

TCM syndrome scores will be assessed according to the Guidance Principle of Clinical Research on New Drug of Traditional Chinese Medicine (2002 edition) [27], which is widely used in the evaluation of TCM syndrome in China. The efficacy is classified into clinical recovery, markedly effective, effective, and non-effective: clinical recovery, TCM clinical symptoms disappeared or symptom score reduction ≥ 95%; markedly effective, symptom score reduction ≥ 70% and < 95%; effective, symptom score reduction ≥ 30% and < 70%; and non-effective, no significant improvement in clinical symptoms of TCM or symptom score reduction < 30%. The calculation formula is based on the nimodipine method: Efficacy index = [(score before treatment – score after treatment) / score before treatment] × 100%. The evaluation will be performed at baseline, day 6, day 21, and day 42.

### Safety assessments

The routine blood panel, urine analysis, liver function test, renal function test, haemagglutination test, and ECGs will be investigated before and after completing all the interventions. Adverse events (AEs) during treatment will be classified according to the US National Cancer Institute, Common Terminology Criteria for Adverse Events v4.0 (NCI CTC 4.0). Once grade 3–4 AEs occur, we will address AEs in a timely manner. All AEs will be observed and documented in detail. All abnormal changes from the baseline laboratory tests will be evaluated.

### Sample size calculation

The Revised Piper Fatigue Scale (using a 0–10 scoring system: higher scores indicate higher severity) score is deemed the primary therapeutic index. The sample size calculation was based on our pilot study, which included 22 patients in the analysis and showed that the RPFS score in the JPSS group was 53.21 ± 23.10 and the RPFS score in the P group was 91.13 ± 44.36 after two courses of treatment. Power Analysis and Sample Size (PASS, version 15, NCSS, 201) software was used to perform the sample size calculation. A sample size of 25 patients in each group was required to detect a significant difference (*p* < 0.05) between the two groups, with a bilateral 5% type I error and a power of 90%, assuming a 20% dropout rate. In summary, a total of at least 50 patients are required. The sample size calculation was performed by the Clinical Research Center, South China Research Center for Acupuncture and Moxibustion, Guangzhou University of Chinese Medicine.

### Data management and quality control

**Fig. 2:**
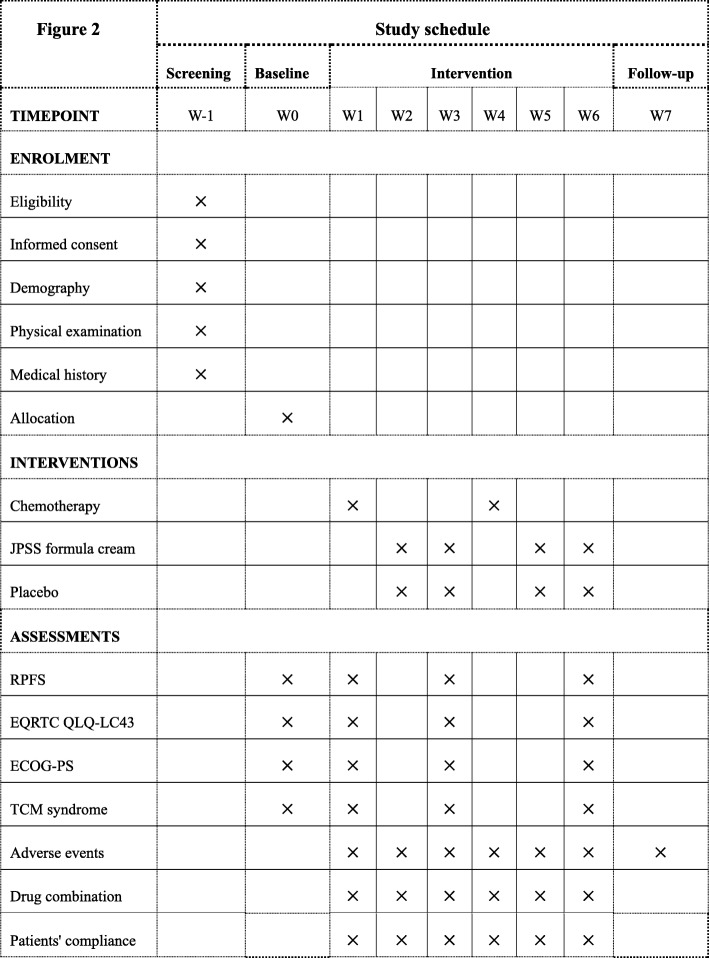
Study schedule

Three research nurses involved in this study have been trained and have obtained corresponding knowledge. One nurse will call each patient every week to monitor compliance with the cream. Moreover, the other two nurses will help with the assessment of the mentioned questionnaires. Whenever there are unusual situations, including possible AEs, the nurses are required to report them to the researcher in a timely manner and record it. A brief diagram of the study schedule is shown in Fig. 2.

### Statistical analysis

The data will be analysed according to the intention-to-treat (ITT) principle. The primary objective is to determine whether the average improvement in CRF from baseline to day 42 in patients receiving JPSS is different from that in patients receiving the placebo. A repeated-measures analysis of variance (ANOVA) will be used to examine the effect of the interventions and time on the RPFS. If the data are normally distributed, the *t* test will be used to analyse the scores for the RPFS, EORTC QLQ-LC43, and TCM syndrome between the experimental group and the placebo group. Stratified analysis according to different chemotherapy schemes and the number of cycles will be used to analyse the differences in the RPFS, EORTC QLQ-LC43, and TCM syndrome scores. The baseline characteristics of patients, including sex, age, body mass index, and histopathological type, between the experimental group and the control group will be compared using the chi-squared test. The number of patients experiencing adverse events (AEs) will be compared between these two groups using the chi-squared test. *p* < 0.05 (two-sided) will be considered statistically significant. All analyses will be performed using SPSS version 22.0.

We will check and analyse the reasons for missing data. The missing values will be estimated using the last observation method (LOCF) [31]. After the main analysis is completed, sensitivity analysis will be performed on each dataset to evaluate the impact of missing data on the test results.

### Ethics statement

This study has been approved by the Ethics Committee of the First Affiliated Hospital of Guangzhou University of Chinese Medicine (approval number: No. ZYYECK[2018]045). All participants will be informed of the details of the study and sign informed consent.

## Discussion

CRF may occur at any time, and it may last for a long period even after cancer goes into remission. Given the high morbidity of fatigue in cancer patients, researchers have put a great deal of effort into the study of the mechanism and treatment of CRF. Despite this effort, there is still no substantial clinical evidence in support of the currently available drugs [32]. Currently, an increasing number of CRF patients choose to use herbal-related products to improve their health conditions, and TCM may become a popular alternative for CRF patients with a broad application perspective [33]. The JPSS herbal formula cream, which is used extensively in our hospital as an internal preparation for CRF, is considered an essential complementary therapy with beneficial effects. Early clinical application and observation have also laid a foundation for its use. Due to the lack of quality randomized placebo-controlled trials at present, we designed this study to evaluate the efficacy and safety of JPSS.

Some studies have shown that TCM herbal products are effective and safe for the treatment of CRF. A prospective trial was conducted to assess Ren Shen Yang Rong Tang (RSYRT) decoction for CRF; fatigue severity decreased significantly before therapy to 6 weeks after therapy, from 7.06 to 3.30 on a 0–10 scale, and no discomfort or toxicity was observed [34]. In another randomized control trial, 40 patients with cancer-related fatigue were randomized into two groups: an oral Bojungikki-tang group or a waitlist group. After 2 weeks, the Bojungikki-tang group showed statistically significant improvements in fatigue level, assessed by the Visual Analogue Scale of Global Fatigue (VAS-F) measuring the severity of fatigue (experimental vs. control: − 1.1 ± 2.1 vs. 0.1 ± 0.9, *p* < 0.05). The results indicated that Bojungikki-tang offers strong evidence in terms of CRF and QoL in cancer patients [35]. Similarly, Lv WJ [36] demonstrated that Yiqi Chutan decoction had an advantage for treating CRF. A total of 83 NSCLC patients were randomized into a treatment group (41 cases) and a control group (42 cases). The treatment group was given Yiqi Chutan Decoction combined with DP/GP, while the control group received DP/GP only. After two treatment cycles, the CRF severity in the treatment group was significantly alleviated compared with that in the control group, with fewer adverse reactions observed [36].

Several preliminary clinical studies have also been conducted to assess the effectiveness of the JPSS formula cream for patients with CRF. A prospective, randomized, controlled study was conducted as already mentioned, which indicated that JPSS might have a remarkable influence on CRF.

We aim to conduct this trial to evaluate the efficacy and safety of JPSS for treating CRF in NSCLC patients and provide high-quality clinical evidence in this area. However, there are some limitations to our study. First, fatigue may persist for a long time after the end of treatment. However, the intervention and follow-up will only last for two courses of chemotherapy, which cannot indicate and assess the long-term efficacy of JPSS during the whole four to six cycles of chemotherapy. A longer intervention and observation period will be our future research direction. Second, to expand the target group, the chemotherapy regimen and the number of cycles will not be required to be the same in this research. Therefore, we will conduct a stratified analysis according to different schemes and the number of cycles. Based on its herbal ingredients and its convenience of use, we consider the herbal formula cream a promising herbal formula that is worthy of being studied and used for chronic diseases, especially for CRF that results in persistent fatigue.

## Trial status

This trial have begun to recruit participants in August 2019 using protocol version 3.0, dated 24 September 2018, and recruitment is expected to be completed in February 2020.

## Data Availability

Not applicable.

## Abbreviations

CRF: Chemotherapy-related fatigue
ECOG-PS: Eastern Cooperative Oncology Group performance status
EORTC QLQ-LC43: the European Organization for Research and Treatment of Cancer Quality of Life Questionnaire LC43
JPSS: Jianpishengsui
NCCN: National Comprehensive Cancer Network
NSCLC: Non-small cell lung cancer
QoL: Quality of life
RPFS: Revised Piper Fatigue Scale
TCM: Traditional Chinese Medicine
